# Harnessing New Tools for Old Challenges: Optimising Neat Plasma Proteomics with Automation and Gas-Phase Fractionation

**DOI:** 10.1021/acsmeasuresciau.5c00166

**Published:** 2026-01-05

**Authors:** Colleen B. Maxwell, Dan Lane, Nikita Bhakta, Emer M. Brady, Richard D. Haigh, Rajinder Singh, Gaurav S. Gulsin, Gerry P. McCann, Leong L. Ng, Donald J. L. Jones

**Affiliations:** † Division of Cardiovascular Sciences and NIHR Leicester Cardiovascular BRC, Glenfield Hospital, 4488 University of Leicester, Leicester LE3 9QP, U.K.; ‡ Leicester van Geest MultiOMICS Facility, Hodgkin Building, University of Leicester, Leicester LE1 9HN, U.K.; § Leicester Drug Discovery and Diagnostics (LD3), Maurice Shock Medical Sciences (MSB), University of Leicester, Leicester LE1 7RH, U.K.; ∥ Division of Cancer Sciences, Leicester Royal Infirmary, LE1 5WW, Robert Kilpatrick Clinical Sciences Building, Leicester, U.K.

**Keywords:** plasma proteomics, timsTOF, evosep, evotip loading, automation, coronary artery disease, DIA-PASEF

## Abstract

Advances in high-throughput mass spectrometry have shifted the bottleneck in plasma proteomics from data acquisition to sample preparation. While enrichment and depletion strategies enable detection of low-abundance proteins, their complexity and cost limit scalability and clinical translation. Targeting midto-high abundance proteins from neat plasma offers a practical, reproducible alternative aligned with clinical workflows. Here, we combine fully automated sample preparation and Evotip loading on the Bravo AssayMAP system with extensive method optimization on the timsTOF HT and gas-phase fractionation deep spectral libraries to advance neat plasma proteomics. Automation reduced hands-on time by 88% and significantly improved robustness. Mixed-mode searching with a 1788-protein library increased identifications by up to 31% at a throughput of 100 samples per day, with less than 15% variation across plates. In a coronary artery disease cohort, we quantified 936 biologically relevant proteins and found 42 dysregulated compared to healthy controls. This streamlined, high-throughput workflow enables deep, reproducible analysis of neat plasma at scale, paving the way for population-level biomarker discovery and clinical implementation.

## Graphical abstract

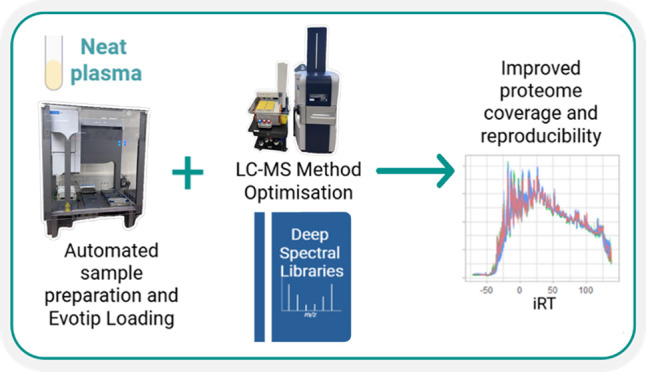

## Introduction

Circulating plasma proteins represent a rich source of clinically actionable biomarkers. Indeed, blood tests remain the most widely used diagnostic tool in medicine; they are minimally invasive, scalable, and cost-effective.^1^ Recent advances in mass spectrometry (MS) and liquid chromatography (LC) have transformed plasma proteomics into a high-throughput discipline capable of analyzing hundreds of samples per day, bringing population-scale proteomic studies on the scale of genomics and transcriptomics within reach.^2^

Next-generation instruments such as the Orbitrap Astral,^3^ ZenoTOF 8600,^4^ and timsTOF HT now offer exceptional sensitivity, mass accuracy, and scan speed. In particular, the timsTOF HT’s data independent acquisition (DIA) parallel accumulation serial fragmentation (PASEF) mode, coupled with short gradient LC systems like the Evosep ONE, enables deep and rapid plasma proteome coverage.^5^ The timsTOF HT’s TIMS-XR analyzer doubles ion storage and accelerates ion mobility ramping, facilitating faster and more comprehensive MS/MS acquisition. Moreover, TIMS permits gas-phase fractionation (GPF), allowing deep spectral libraries to be generated without time-consuming offline fractionation, greatly enhancing proteome depth with minimal sample handling.^6^ While GPF has been demonstrated for cell lysates and depleted plasma,^7–9^ its application to neat plasma remains unexplored.

Despite these advances, plasma remains an exceptionally challenging matrix, with protein concentrations spanning over 10 orders of magnitude and dominated by high-abundance species such as albumin.^10^ Plasma also exhibits wide heterogeneity across individuals and is susceptible to massive preanalytical variability if sample collection steps including are not tightly controlled within studies.^11^ Balancing the enrichment of low-abundance proteins against workflow simplicity and reproducibility to avoid adding even more technical variability remains a central challenge for clinical translation. Deep-coverage strategies continue to push depth of coverage into the ng/L abundance range, such as extensive fractionation, novel extracellular vesicle isolation techniques, and high abundance protein depletion with acid or organic solvents, extensively evaluated elsewhere.^12,13^ These strategies hold great value in terms of unravelling novel pathways and increasing understanding of pathophysiological processes, but can be difficult to scale to population studies due to cost, complexity, and sample volume requirements.

The clinical community requires reproducibility and ease of measurement to support actionable clinical decisions. Therefore, focusing on midto-high abundance proteins obtained through straightforward neat plasma processing may hold more promise. Indeed, integrating panels of a few hundred midto-high abundant proteins across large population studies, particularly when leveraged with artificial intelligence models, may provide the necessary power to support early disease detection and prognosis.^14^ In particular, for cardiovascular and metabolic disorders, many apolipoproteins, markers of inflammation, and coagulation factors are readily accessible within the top few hundred most abundant proteins.

Recent automation advances promise reproducible and scalable plasma proteomics. Platforms such as AutoMP3,^15^ the Opentrons OT-2,^16^ and Geyer’s automated workflow using blood droplets^14^ demonstrate the feasibility of fully automated, end-to-end sample preparation. Here, we report the first use of fully automated Evotip loading with the Bravo AssayMAP platform and GPF-derived deep spectral libraries to advance neat plasma proteomics. Combined with extensive DIA-PASEF optimization on the timsTOF HT, this workflow achieves deep, reproducible proteome coverage of neat plasma at high throughput.

## Methods and Materials

### Experimental Design

The study was designed in four phases to assess robust, scalable neat plasma proteomics. First, we benchmarked fully automated Evotip loading on the Bravo AssayMAP against manual loading and an automated variant with solvent B prerinse (*n* = 16 per condition), assessing protein group coverage, data completeness, %CV, ppp, and hands-on time. Second, we optimized DIA-PASEF methods on the timsTOF HT using Evosep ONE 100 SPD by systematically varying ion mobility and mass ranges, ramp/accumulation times, window number/widths (including py_diAID variable windows) and diagonal-PASEF, and by comparing Endurance versus Performance columns to balance depth with sufficient ppp for quantitative precision (*n* = 6 per method). Third, we assessed longitudinal reproducibility of the end-to-end sample preparation workflow across three independent 96-well plates (*n* = 54 total), distributing pooled plasma in carefully selected positions to ensure any intraplate variation would be accounted for. In parallel, we generated a deep hybrid library from fully automated high-pH RP fractionation and DIA/DDA gas-phase fractionation, then performed comparisons of library-only, FASTA-only, and mixed-mode searches in Spectronaut using *n* = 16 and *n* = 54 pooled plasma samples. Finally, in a matched case–control cohort (45 coronary artery disease, 45 matched controls), we applied the final method and tested differential expression using BH-adjusted *p*-value <0.05 and fold-change >1.5, followed by pathway enrichment.

### Plasma Collection

Plasma was obtained from the biomedical research informatics centre for cardiovascular sciences (BRICCS) cohort, collected with informed consent under research ethics committee (REC) reference: 09/H0406/114. Blood was collected by venipuncture into tubes containing ethylenediaminetetraacetic acid (K_2_EDTA) anticoagulant and stored on ice until centrifugation. The blood was centrifuged at 3200 rpm for 20 min at 4 °C using a Sorvall ST 8 Small Benchtop Centrifuge (Thermo Scientific, Loughborough, U.K.). The centrifuge was allowed to come to a halt without assisted deceleration, and after stopping, the plasma was harvested from the top of the tubes, ensuring the pipet tip did not come within 3 mm of the buffy coat layer of white blood cells and platelets. The plasma was stored at – 80 °C until analysis. Propensity score analysis was used to balance and select 45 cases with coronary artery disease and 45 healthy matched controls based on a 1:1 matching algorithm. Cases were defined by having a coronary artery calcium score (CACS) ≥ 200 and controls CACS <10. Baseline characteristics of the two matched groups are shown in Supporting Table S1.

### In-Solution Digestion

Aliquots of plasma were defrosted at room temperature. A volume of plasma corresponding to 100 μg protein (approximately 1–2 μL) for each sample was determined by bicinchoninic acid assay and aliquoted into a twin.tec 96-well plate (Eppendorf, Hamburg, Germany). Samples were made up to 10 μL with 50 mM ammonium bicarbonate (pH 7.8). Sample preparation for bottom-up proteomics was performed using the “In-solution Digestion” workflow on the AssayMAP Bravo Platform (Agilent Technologies, Santa Clara, CA, USA). Each step was performed with 15 mix cycles and 3 wash cycles. To the 10 μL starting volume, 20 μL denaturant mixture was added to each sample (2 μL 75 mM dithiothreitol (DTT), 3 μL 1% rapiGest (Waters, Milford, CT, USA), 15 μL 50 mM ammonium bicarbonate pH 7.8). The plate was sealed and incubated off-deck in a ThermoMixer C (Eppendorf, Hamburg, Germany) at 65 °C for 30 min with agitation at 350 rpm. The plate was returned to the deck and allowed to cool to room temperature, then iodoacetamide (IAA) was added to a final concentration of 10 mM followed by incubation with lid for 30 min. Ammonium bicarbonate (80 μL) was added followed by Trypsin/Lys-C (Promega, Southampton, UK) at a ratio of 1:25 (protease/protein). The plate was sealed and incubated off-deck at 37 °C for 16 h with agitation at 350 rpm. Samples were acidified with formic acid (FA) 10% added to a final concentration of 1% (v/v). The plate was centrifuged at 4000*g* for 25 min using an Eppendorf Centrifuge 5810R (Eppendorf, Hamburg, Germany), and the supernatant collected and transferred into a twin.tec 96-well plate.

### Peptide Clean-up

Clean-up was performed on the AssayMAP platform following the “Peptide Clean-up” workflow with AssayMAP 5 μL C18 cartridges (Agilent Technologies, Santa Clara, CA, USA) following the default parameters for wash cycles. Cartridges were primed with 100 μL 90% acetonitrile (MeCN) at a flow rate of 300 μL/min and equilibrated with 50 μL 0.1% FA at a flow rate of 10 μL/min. Digests (100 μL) were loaded at a flow rate of 5 μL/min followed by a 25 μL cup wash cycle and 50 μL internal cartridge wash with 0.1% FA at 10 μL/min. Peptides were eluted with 25 μL 70% MeCN +0.1% FA into polypropylene U-bottom 96-well plates (Greiner, Kremsmünster, Austria) at a flow rate of 5 μL/min, dried in vacuo and stored at – 80 °C until required.

### High pH Reversed-Phase Fractionation

Dried plasma peptides were resuspended in 100 μL of 25 mM ammonium formate (pH 10) and subjected to high pH reversed-phase (RP) fractionation with the AssayMAP Bravo platform using the “Fractionation” workflow with AssayMAP 5 μL RP-S cartridges (Agilent Technologies, Santa Clara, CA, USA). The cartridges were primed with 100 μL 90% MeCN at a flow rate of 300 μL/min followed by equilibration with 50 μL 25 mM ammonium formate at 10 μL/min. Samples were loaded at 5 μL/min followed by a 25 μL cup wash cycle and 50 μL internal cartridge wash with 25 mM ammonium formate at 10 μL/min. The peptides were eluted in 7 fractions with 25 mM ammonium formate, using increasing concentrations of MeCN (5, 10, 15, 20, 30, and 80%). The fractions were dried in vacuo and stored at – 80 °C until analysis.

### Evotip Loading

Peptide concentration was determined by *o*-phthalaldehyde (OPA) assay (Pierce Quantitative Fluorometric Peptide Assay; Thermo Scientific, Loughborough, U.K.) and peptide normalized to 600 ng was loaded onto Evotips (Evotip Pure, Evosep, Denmark) following either the manual or fully automated Evotip loading protocol as specified per experiment. Manual Evotip loading was performed following the manufacturer’s protocol (PR-001D). Briefly, Evotips were washed with 20 μL solvent B (MeCN) and centrifuged with an appropriate balance box at 800 g for 60 s. Evotips were soaked in propanol until all tips were pale white and equilibrated with 20 μL solvent A (0.1% FA), then centrifuged at 800*g* for 60 s. Normalized samples were loaded in 20 μL followed by centrifugation at 800*g* for 60 s. Evotips were washed with 20 μL solvent A, then centrifuged at 800*g* for 60 s, followed by a final step to keep the tips wet by transferring 100 μL solvent A, then centrifuging at 800*g* for 10 s. Solvent A was added to the box containing the Evotips.

Automated Evotip loading was performed using the Agilent Bravo AssayMAP, as per the Agilent and Evosep step-by-step guides (IN-003A). The adaptors were purchased from Fintiede Solutions (Glasgow, UK) as part of the Agilent AssayMAP Bravo loading kit. Residual wash liquid was removed from the AssayMAP tips and the appropriate number of Bravo 250 μL tips (Agilent) were set up using the integrated automated tip setup (deck positions 5 and 6). The Agilent tip loading station was adapted by adding Evotip tool holder and placing the Evotip 96-head sealing mat and Evotip 96-head plate within it. The tool holder was tightly screwed into position (deck position 2). Normalized samples (30 μL) were aliquoted into an Eppendorf twin.tec 96-well plate (deck position 4), enabling 20 μL to be loaded robustly without reaching the plate dead volume. The appropriate number of Evotips were placed onto a Universal Evotip Adaptor which was secured to a 96-well deep pyramid base reservoir (deck position 7). Propanol was added to a single-well plate which was fitted with a universal Evotip adapter (deck position 8). Solvent A was transferred to a 96-well pyramid base reservoir (deck position 9). Following deck setup, shown in Supporting Figure S1B, the protocol was automatically executed within VWorks software. During the protocol, solvent A was transferred to the tips while seated on deck position 7, followed by the sample, and then more solvent A, with each layer being separated by air gaps (Supporting Figure S1A). The Evotip rack was transferred to the propanol rack at deck position 8 to allow conditioning, then transferred back to position 7. The AssayMAP then picked up the head plate (secured with the sealing mat) and utilized positive air pressure to push the layers through the sorbent bed, leaving residual solvent A to ensure the tips remained wet.

### Liquid Chromatography–Mass Spectrometry

Samples were subjected to LC–MS/MS analysis using the Evosep ONE LC (Evosep Biosystems, Odense, Denmark) coupled to a timsTOF HT mass spectrometer (Bruker Daltonics, Bremen, Germany). The LC was operated with a 100 SPD standard 11.5 min analytical gradient using the EV1109 Performance analytical column ReproSil Saphir C18, 1.5 μm beads by Dr Maisch, 8 cm × 150 μm. The Endurance column (reproSil-Pur C18, 3 μm beads by Dr Maisch. Eight cm × 100 μm, EV1064) was also assessed under the same LC conditions. Mobile phase A was 0.1% FA, mobile phase B was MeCN +0.1% FA. The PASEF windows used during initial protocol optimization, including py_diAID, DIAGONAL-PASEF, are described in full in the Supporting Information timsTOF HT methods. For the final optimized method, DIA-PASEF scan mode was used with a custom optimized PASEF windows. The scan range set at 392–1017 *m*/*z*, the TIMS mobility range to 0.67–1.3 V cm^–2^, and ramp and accumulation times were both set to 60 ms. The method included 15 DIA-PASEF scans with two 25 Da windows per ramp, resulting in an estimated cycle time of 1.06 s. Gas-phase fractionation for library generation was performed according to three schemes: (1) the DIA-PASEF GPF scheme developed by Penny et al.,^8^ comprising seven fractions; (2) the DIA-PASEF GPF scheme (scheme A) developed by Rice and Belanil.^9^ comprising five fractions; (3) the four range DDA-GPF scheme from Guergues and colleagues.^7^ Data was acquired using Compass HyStar version 6.3 with real-time processing in ProteoScape version 2025b.

### Raw Data Processing and Statistical Analysis

Raw data were processing using Spectronaut version 20.2.250922.92449 (Biognosys, Zurich, Switzerland).^17^ Database searching was performed against the *Homo sapiens* reviewed FASTA using directDIA mode with BGS factory settings using enzyme cleavage rules for Trypsin/P, LysC. All statistical analyses were carried out using R version 4.5.0 (The R Foundation for Statistical Computing) on RStudio 2023.03.0 build 386 (RStudio, Inc., Boston, MA). Stratified single hit protein false discovery rate (FDR) was used for protein identification, with FDR set to 1%. For the differential expression analysis, data preprocessing was carried out using the R packages NOISeq^18^ and Caret^19^ to filter features which were not statistically relevant from the data set, normalize the data, impute zero values, and assess and remove sources of unwanted technical variation. Differential expression analysis was carried out using the R package Limma.^20^ The results were filtered for proteins with Benjamini-Hochberg FDR-adjusted *p*-values <0.05 and log_2_(FC) > 0.58 (FC > 1.5). Metascape was used for gene ontology (GO) overrepresentation tests.^21^

## Results

### Evaluation of Automated Tip Loading

The fully automated Evotip loading protocol using the Bravo AssayMAP was compared to the standard manual Evotip loading protocol. The manual Evotip loading protocol begins with prerinsing the tips with MeCN (solvent B) prior to conditioning with IPA, which is omitted from the AssayMAP protocol, thus we also performed automated Evotip loading with an additional rinse step with solvent B to assess any differences arising from the omission of this step. Plasma samples prepared using the in-solution digestion and peptide cleanup on the AssayMAP were pooled to eliminate sample preparation as a source of variability and 600 ng were loaded onto 16 Evotips (the first two adjacent columns) following either the manual protocol, the fully automated AssayMAP protocol, or the automated protocol + prerinse with solvent B. The proteome coverage, reproducibility, data completion and time taken to perform the Evotip loading were assessed for each technique (Figure 1). Data was acquired using the 100 SPD standard Evosep ONE gradient with the EV1064 endurance column and default DIA-PASEF settings as described in the Methods and Materials. Full lists of the proteins identified in this experiment and throughout are available in Supporting Information-Protein IDs.

**Figure 1:**
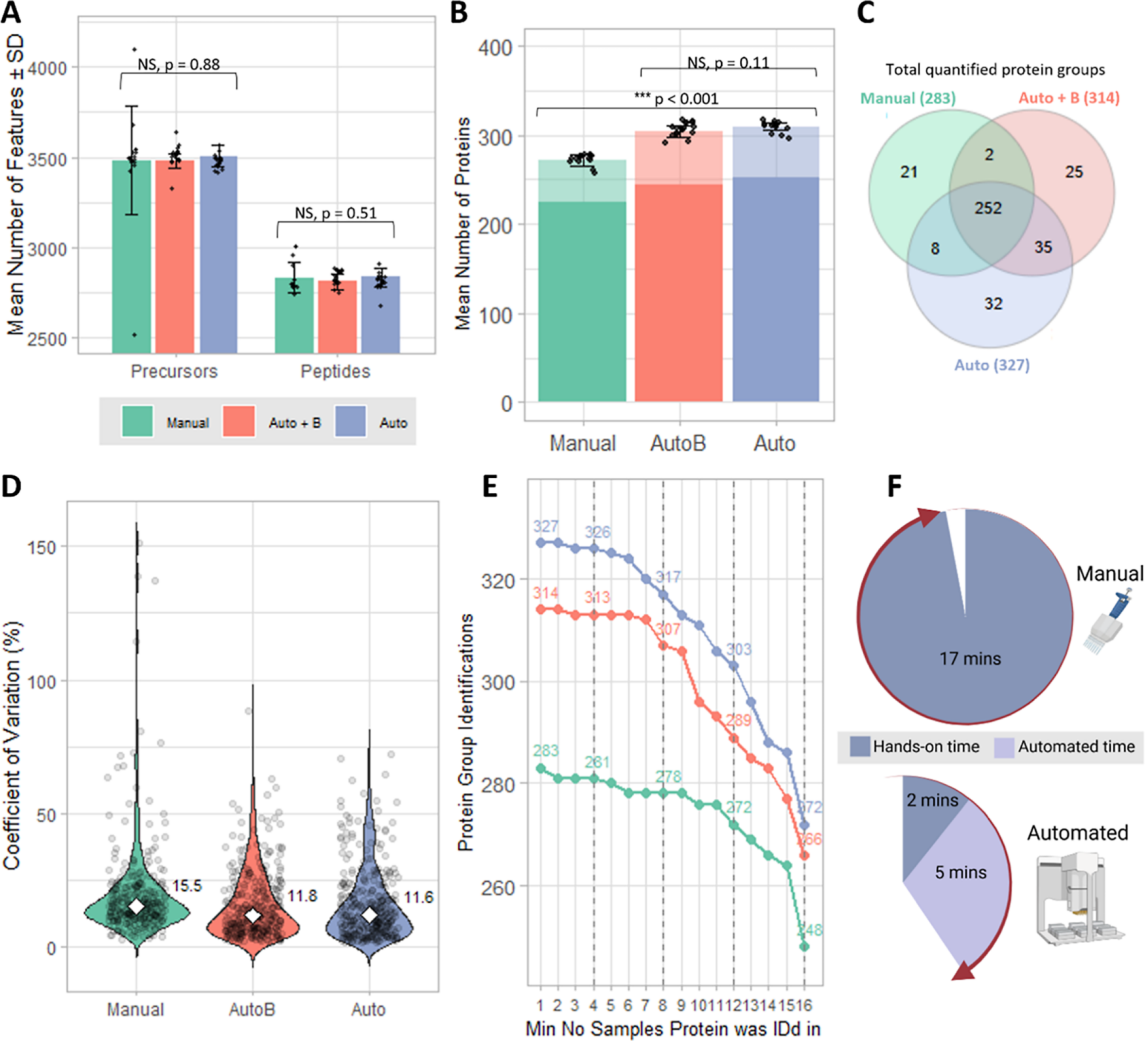
Comparison (*n* = 16) of manual Evotip loading (“Manual”, green) to the fully automated loading protocol using the Bravo AssayMAP (“Auto”, blue) and the automated protocol with additional prerinse with solvent B (“AutoB”, pink). (A) Mean number of precursors and peptides identified in each sample per method, ± σ. The statistical significance of differences between the three groups (ANOVA) is shown. (B) Mean number of proteins groups identified in each sample per method, ± σ. Proteins which were identified based on one peptide hit are translucent. The statistical significance of differences between the groups (ANOVA) is shown. (C) Venn diagram of the total number of quantified proteins for each method and the overlap between these. (D) Violin plot of the % CV distribution across the 16 samples for all quantified proteins for each method. The median CV is indicated with the white marker and is annotated to the right of each violin. (E) Data set completeness across the 16 samples for each method. (F) Pie charts showing the time take (hands-on and automated) to perform the manual and automated methods. Created in BioRender https://BioRender.com/hzog0yz.

There was no significant difference in the mean number of precursors, peptides, or protein groups per sample (Figure 1A,B) between the automated protocol and the automated protocol + solvent B prerinse. In terms of the total quantified protein groups across all samples (Figure 1C), there were more protein groups identified overall in the automated protocol (327) compared to the automated protocol + B(314), with 82% overlap in the protein groups. There was also a similar %CV distribution (Figure 1D) with very close median %CVs of 11.6 (automated) and 11.8 (automated + B). The data also demonstrated very similar level of data completeness (Figure 1E). Thus, the omission of the MeCN prerinse in the automated protocol did not make any significant difference to the reproducibility or data completeness, with a slight increase in total quantified protein groups observed.

There was no significant difference in the mean number of precursors or peptides identified in the automated protocol compared to the manual protocol (Figure 1A), however there were significantly more protein groups identified on average per sample in the automated protocol (310 compared to 272, Figure 1B), as well as more proteins in total (327 compared to 283, Figure 1C). Single peptide hit proteins consistently accounted for 21–22% of IDs across the methods. Additionally, the manual protocol exhibited the largest variation across samples for precursors, peptides, and proteins, as can be seen by the ± σ bars. Data completeness was high for all methods, as can be seen in Figure 1E. The lower reproducibility of the manual protocol can also be seen by the %CV distribution and higher median %CV of 15.5 (Figure 1D). For an experienced researcher to perform the protocol, we determined it to took an average of 17 min of hands-on time to perform the manual Evotip loading protocol (*n* = 16 tips) using a multichannel pipet, whereas the automated protocol required 2 min manual setup time, and 5 min automated time to complete (Figure 1F), thus resulting in a decrease in hands-on time of 88%, in addition to no requirement for a balance tip box as is required for centrifugation.

### Optimisation of LC–MS/MS Method

After establishing the automated Evotip loading protocol with the Bravo AssayMAP as more reproducible and achieving greater protein group coverage compared to the manual protocol, we sought to further increase the depth of neat plasma coverage by optimization of the LC–MS/MS parameters. DIA-PASEF optimization has previously been demonstrated for neat plasma by Metatla et al. using the timsTOF Pro,^22^ and we now take advantage of the faster scanning speed of the timsTOF HT to enable even smaller window widths to be utilized. We focused on changing the DIA-PASEF windows, altering the ion mobility range, the mass range, the number of MS/MS ramps, the number of MS/MS windows, the mass width, the accumulation time (tims1), and ramp time (tims2) to maximize depth of coverage while maintaining a low enough cycle time to achieve an acceptable number of data points per peak (ppp) (at least 5–7) for better quantitative precision.^23^ We also used py_diAID for automated design of PASEF methods with variable window widths and to evaluate diagonal-PASEF, specifically synchro-PASEF, for neat plasma analysis.

The first iteration of method evaluation and optimization was performed using the Evosep EV1064 Endurance column. Briefly, the evaluated methods comprised: (1) the Bruker default QC DIA-PASEF method, “Default”; (2) the Bruker default short gradient DIA-PASEF method, “Default Short”; (3) an optimized method with broader mobility inspired by the work of Metatla et al. on the timsTOF Pro,^22^ “Optimized 1”; (4) second optimized method with narrower windows and more MS/MS ramps, “Optimized 2”; (5) a method with variable window widths designed using py_diAID, “Py_diAID”,^24^ and; (6) diagonal-PASEF acquisition mode, specifically synchro-PASEF,^25^ “Diagonal”. Our aim was to maximize proteome coverage while retaining robustness, which we assessed by measuring the ppp and reproducibility (% CV) across *n* = 6 pooled plasma samples. The mass and mobility ranges, precursor and peptide coverage, cycle times, % CV and data completeness are summarized for each method in Supporting Table S2. The PASEF windows are provided in full in Supporting Information timsTOF HT methods.

The mean number of proteins identified per sample, total quantified proteins and ppp are shown in Figure 2A. The Default and Default Short methods achieved similar proteome coverage and mean ppp, identifying 288 and 290 protein groups with mean ppp of 6.9 and 6.7, respectively. However, the Default method identified more peptides (2891 vs 2462) and had a lower median % CV (7.4 vs 9.6). The first optimized method, inspired by Metatla et al., used a wider mass and mobility range and more MS/MS ramps,^22^ increasing coverage to 3072 peptides and 302 protein groups (median %CV 8.0), with a reduced mean ppp of 6.2. We slightly reduced the mass range and performed further optimization to take advantage of the faster scanning speed of theHT, decreasing the window mass width and increasing the number of MS/MS ramps and windows, achieving the highest coverage (3274 peptides, 326 protein groups, median %CV 8.5), though with a lower mean ppp of 5.8. We also tested py_diAID for automated optimization and diagonal-PASEF acquisition. These yielded faster cycle times (0.77 and 0.42 s), higher ppp (8 and 11), and %CVs of 8.4 and 6.7, respectively, but lower coverage (272 and 261 protein groups). Overall, the Optimized 2 method gave the greatest coverage, while diagonal-PASEF offered the highest ppp and lowest %CV. Proteins identified using stratified single hit protein FDR accounted for between 15–21% of IDs across the methods.

**Figure 2:**
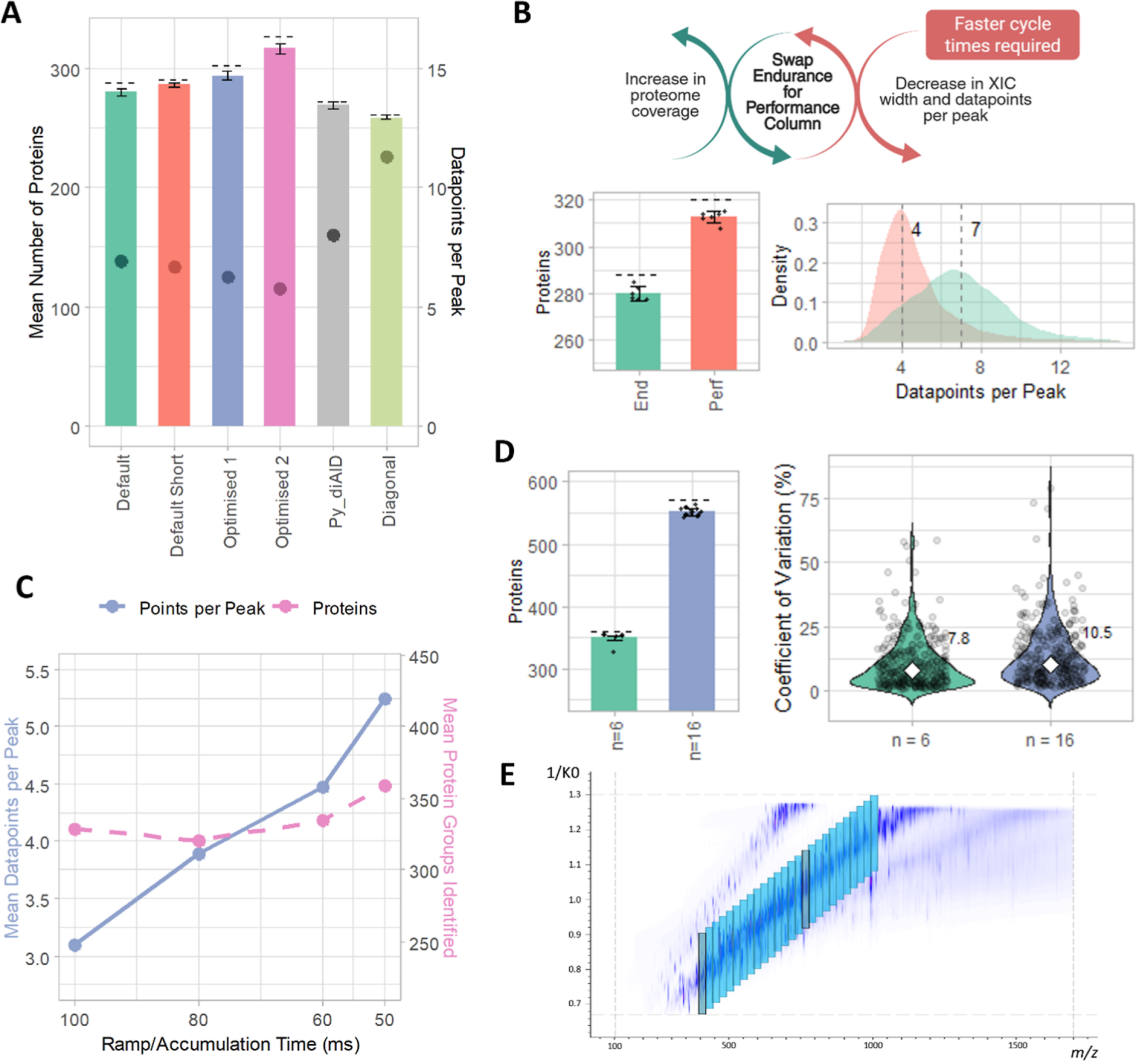
Iterative optimization of DIA-PASEF parameters using pooled neat plasma (*n* = 6) to maximize proteome coverage while maintaining acceptable ppp. (A) Mean number of proteins groups identified in each sample per PASEF method, ± σ. Total proteins indicated by dashed line. Mean number of ppp are indicated by the dot corresponding to the secondary *y*-axis. (B) Effect of swapping the Endurance column for the Performance column on the number of protein groups identified and the distribution of data points per peak. The median ppp is indicated by the dashed lines. (C) The increase in ppp (primary *y*-axis, purple) and protein groups identified (secondary *y*-axis, pink) observed with decreasing the ramp/accumulation time. (D) Number of protein groups and violin plot of the % CV distribution across *n* = 6 samples vs *n* = 16 samples for all quantified proteins for the optimized DIA-PASEF method. (E) The PASEF windows overlaid onto the sample ion cloud for the final optimized DIA-PASEF method.

To optimize LC performance for neat plasma, we then switched from the Evosep 100 SPD method using the Endurance analytical column to the equivalent Performance column (Figure 2B). This is packed with smaller C18 particles, 1.5 μm compared to 3 μm for Endurance, providing greater separation efficiency with sharper peaks. The result is an increase in coverage, for the Default method an increase to 320 protein groups, however the decrease in median extracted ion chromatogram (XIC) width from 1.0 to 0.6 min resulted in a decrease in ppp to 4.71 on average and an increase in % CV to 8.5 (Figure 2B). The Optimized 2 method was similarly affected, with an increase in peptide coverage to 3393 and protein coverage to 329, but a decrease in mean ppp to just 3.1. Lower cycle times were required to achieve acceptable ppp. We achieved this by adjusting the ramp/accumulation times (in synchronization to maintain the highest possibly duty cycle). The mass and mobility ranges, precursor and peptide coverage, cycle times, % CV and data completeness are summarized for each method in Supporting Table S3. Figure 2C explores the Optimized 2 method and how decreasing the ramp/accum time from 100, to 80, 60, and finally 50 ms, decreased cycle time from 1.7 to 0.9 s, and increased mean ppp from 3.1 to 5.2, and quantified protein groups to 359. There was also a decrease in median % CV from 8.7 to 7.8 (Supporting Table S3).

With its low cycle time of 0.42 s, we also evaluated diagonal-PASEF with the Performance column. This achieved the highest ppp of 8 and slightly improved % CV of 6.6, but with reduced coverage of 316 proteins (Supporting Table S3). The optimized 2 method with ramp/accumulation 50 ms was selected for sample analysis for the remainder of the work presented, referred to as the “final optimized method” (Figure 2E). To determine the performance of the optimized method on a larger number of samples, particularly with the advantage of match-between-runs when analyzing larger sample groups, we repeated the analysis for *n* = 16 samples and found an average of 551 protein groups identified per sample, with a total of 570 groups across all 16 samples (Figure 2D). The % CV increased as expected, but remained low with a median of 10.6. Proteins identified using stratified single hit protein FDR accounted for between 14–18% of IDs across the methods.

We also compared the performance of the final optimized method at a gradient of 60 SPD (Supporting Figure S3, Supporting Table S4). Compared to 100SPD, for an increase in gradient time of 82.6% to 21 min, there was an increase in XIC width to 0.8 min and median data points per peak to 6.7. However, this resulted in just a 15% increase in precursors and peptides and a 19.8% increase in the number of protein groups identified to a total of 430. There was also an increase of %CV to 9.7%. Given the relatively small gains in proteome depth for a comparatively large decrease in throughput, we performed the remainder of the analyses at a throughput of 100 SPD.

### Longitudinal Reproducibility of End-to-End Workflow

We evaluated the longitudinal reproducibility of the workflow. For prior experiments, plasma prepared as described using the in-solution digestion and peptide cleanup on the AssayMAP was pooled prior to Evotip loading. For this experiment, neat plasma was pooled at the beginning of the experiment, and aliquoted into a 96-well plate, such that total variation of the end-to-end workflow from all sources could be measured: in-solution digestion, peptide cleanup, automated Evotip loading, and data acquisition using the final optimized DIA-PASEF method, all of which are described in the Methods and Materials. To assess longitudinal reproducibility without requiring the sample and reagent resource of a whole sample plate while still accounting for intraplate variation,^26^ 18 pooled samples were distributed across a 96-well plate to cover corner, edge, and center wells (Figure 3A). To assess interplate variation, the end-to-end workflow was performed for three separate plates with the described sample distribution across 3 days. The plate layout and number of protein groups identified per each sample well across the three plates is shown in Figure 3A, with no visible column-, row-, diagonal-, or edge-based effects observed.^26^

**Figure 3:**
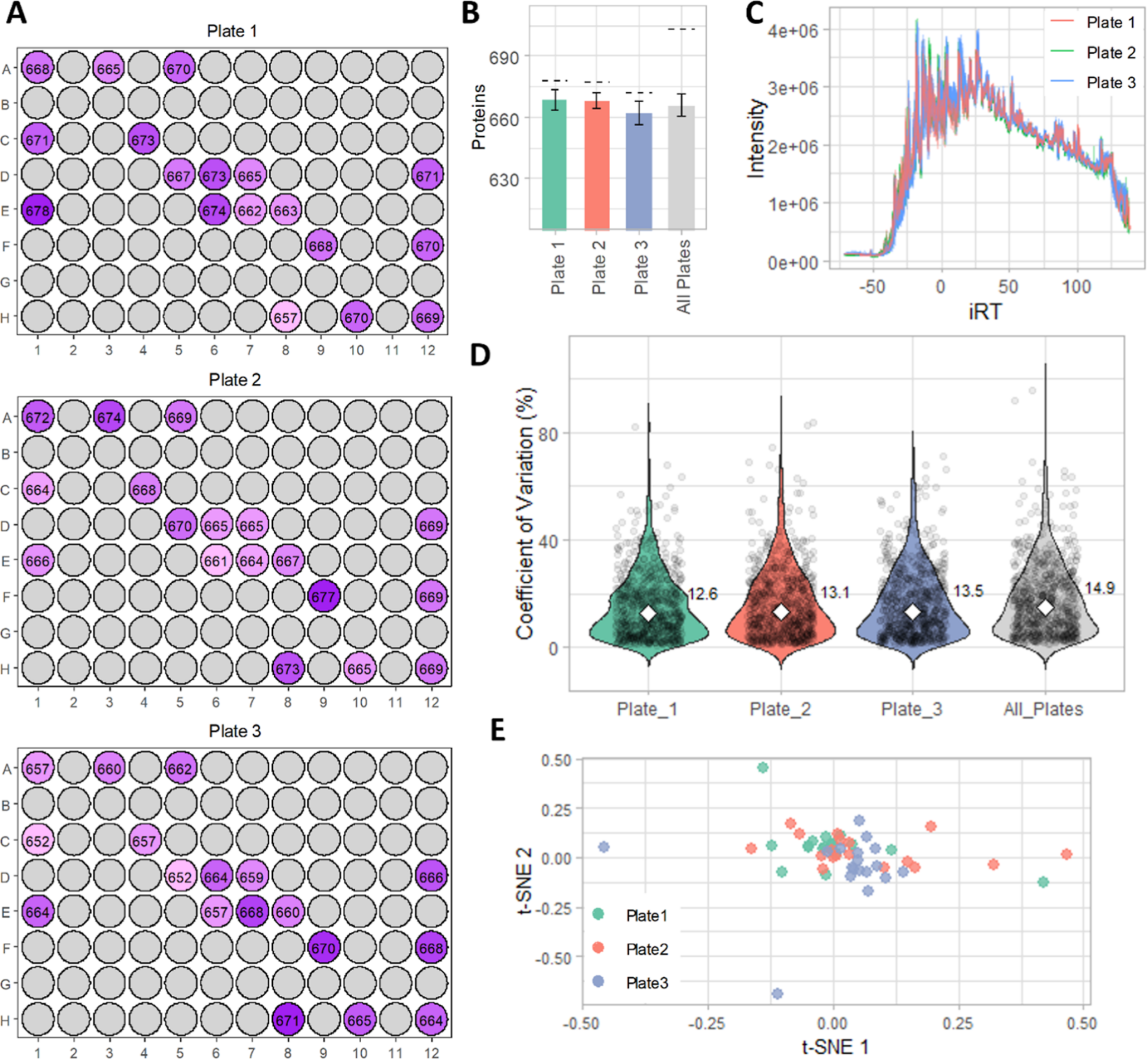
Assessment of the longitudinal reproducibility of the end-to-end optimized, automated workflow. (A) Number of protein groups identified per each well containing plasma across the three plates measured; shading relates to number identifications. (B) The average number of protein groups per sample identified per plate ± σ, and across all plates (gray). The total number of protein groups identified is indicated by the dashed line. (C) Overlaid total ion count (TIC) chromatograms vs indexed retention time for each of the samples, color coded by plate. (D) Violin plot of the % CV distribution across *n* = 18 samples for all quantified proteins for each plate, and for *n* = 54 samples across all three plates (gray). The median CV is indicated with the white marker and is annotated to the right of each violin. (E) t-SNE plot showing clustering of the 54 plasma samples across the three sample plates.

On average, there were very similar average (both 668) and total (678 and 677) protein groups identified in plate 1 and 2, and a slightly lower number in plate 3 (662 average, 671 total) (Figure 3B). In total across the 54 samples in three plates, there were 695 protein groups identified. The overlaid chromatograms for all samples color-coded by plate, shown in Figure 3C, show little variation between runs. The intra- and interplate %CV remained low, with a median of 13.5 or lower in each individual plate, and a %CV of under 15 across all 54 samples (Figure 3D). Using t-SNE to visualize the protein profiles, the three plates show substantial overlap, indicating no major batch effects attributable to plate (Figure 3E). A slight separation of plate 3 from plates 1 and 2 is observed.

The total time for sample preparation (Supporting Figure S2) per 96-well plate was equal to 20.1 h assuming an overnight digestion is used, and this can be multiplexed to 4 plates (totalling 384 samples) if the “Multi-Plate” version of the Bravo AssayMAP in-solution digestion protocol is employed, although this will quadruple the time required for peptide cleanup (1 to 4 h) and Evotip loading (7 to 28 min). Additionally, although we did not demonstrate this, a reduction in digestion time to 4 h would allow the whole protocol to be performed in 8.6 h. Some hands-on time is required to conduct the protocol, principally reagent preparation and transfer to plates (approximately 1 h), setting up the Bravo deck for digestion, cleanup and Evotip loading (approximately 10 min total), and transfer of the plate to the speed vac (5 min).

### Library Generation

Following optimization of the data acquisition method and full evaluation of the sample preparation workflow, we sought to optimize data processing by developing a neat plasma spectral library. TIMS GPF may be used to create deep spectral libraries, which have previously been demonstrated using both DIA- and DDA-PASEF to improve proteome depth in HeLa cell lysate and Top 14 depleted plasma.^6–9^ However, these have not yet been leveraged to increase proteome depth using only neat plasma samples. We performed extensive deep fractionation, incorporating two DIA-PASEF GPF schemes, one DDA-PASEF GPF scheme, and offline high pH RP fractionation, fully automated using the Agilent Bravo. The GPF schemes and offline fractionation method are described in the Experimental Procedures. Mass spectra for the high pH RP fractions were acquired using the final optimized method previously described.

The method which resulted in the largest library in terms of both precursors (6818) and protein groups (1126) was the automated offline high pH RP fractionated library comprising 7 fractions from neat plasma (Figure 4A). The DIA-PASEF GPF scheme developed by Penny et al.,^8^ comprising 7 fractions, contained 875 protein groups and 6638 precursors. The GPF scheme developed by Rice and Belani.^9^ comprising 5 fractions contained 749 protein groups and 6381 precursors. Finally, a DDA-GPF library from Guergues and colleagues was also evaluated,^7^ which as expected achieved a lower proteome depth than the DIA schemes, with 227 proteins from 3020 precursors. A deep, hybrid neat plasma library constructed by combining the four libraries achieved 10767 peptides corresponding to 1788 protein groups. While there was substantial overlap between the proteins contained within each library, each contributed unique proteins to the deep library (Figure 4B). In particular, the offline high pH RP fractionated library contributed 627 unique proteins, while Penny and Rice’s GPF libraries contributed 336 and 236 unique proteins, respectively.

**Figure 4:**
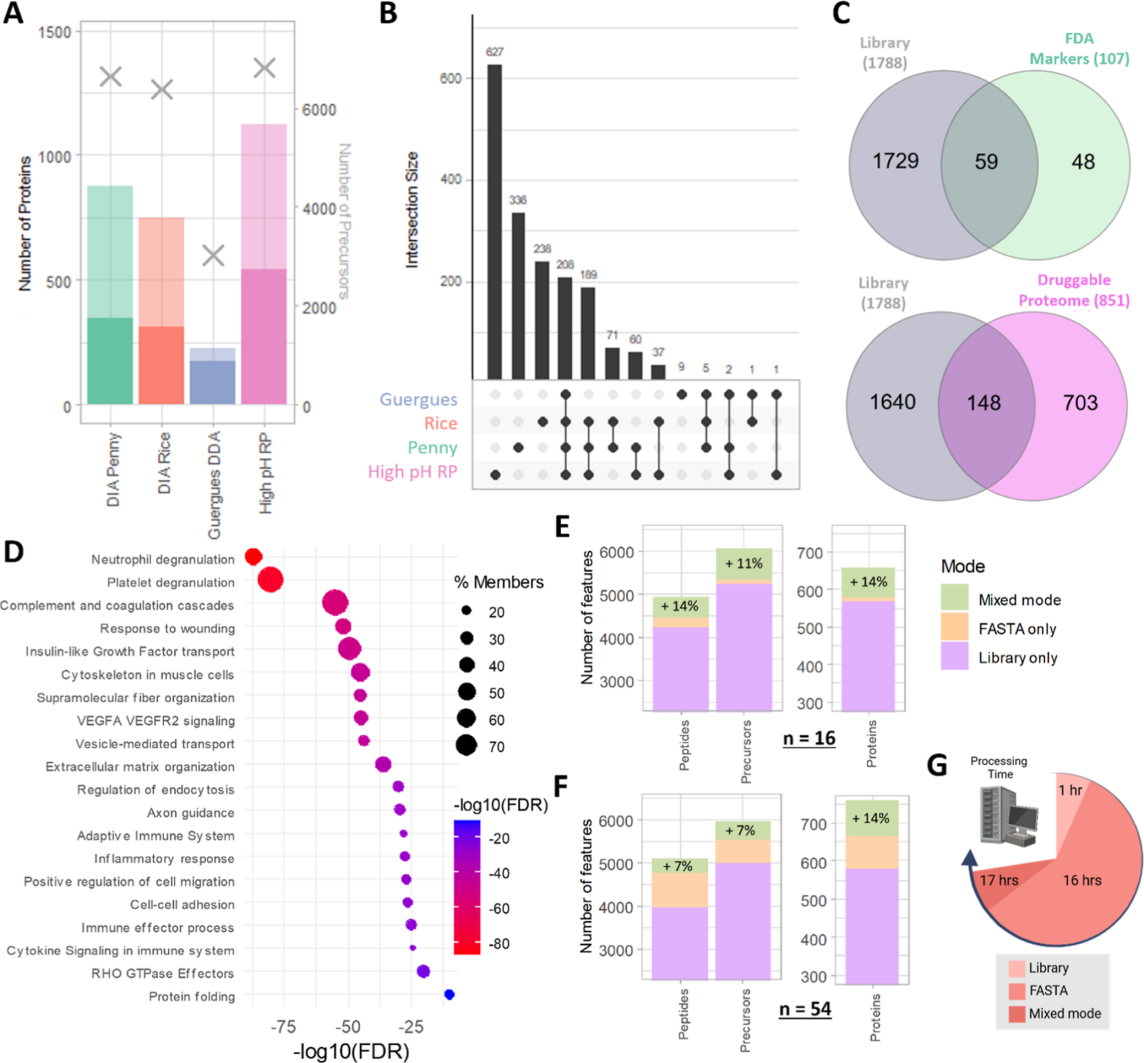
MS/MS library generation using gas phase fractionation and offline high pH reverse-phase fractionation. (A) Number of proteins groups (primary *x*-axis) and precursors (secondary *x*-axis) identified in each library, with single hit proteins indicated by the translucent bars. (B) Upset plot showing the overlap in proteins identified across the four library types evaluated. (C) The number of proteins from the FDA-approved protein biomarkers and druggable proteome which are found in the combined deep library, containing 1788 proteins. (D) Pathways enriched in the combined deep plasma library. (E) The increase in protein IDs gained when searching in mixed mode compared to library-only and FASTA-only for *n* = 16 assayMAP sample prep replicates (single plate reproducibility experiment). (F) The increase in protein IDs in mixed mode for *n* = 54 replicates (longitudinal reproducibility experiment). (G) The search time taken (nearest hour) in Spectronaut for library-only, FASTA-only, and mixed-mode searches for *n* = 54 replicates. Created in BioRender https://BioRender.com/hzog0yz.

The deep, hybrid neat plasma library contained markers of substantial value to plasma biomarker investigations, representing the clinical plasma proteome (Figure 4C). Fifty-nine proteins were present from the small number of FDA-approved clinical assays for proteins in plasma and serum,^27^ as were 148 proteins from the druggable proteome.^28^ We performed pathway enrichment analysis using Metascape^21^ to identify which biological pathways were over-represented in the library (top 20 Figure 4D, top 90 Supporting Figure S4). Pathways were represented with relevance to several disease areas including inflammation, infection and autoimmunity (neutrophil and platelet degranulation, complement, wounding, inflammatory response, cell migration, cytokine signaling), cardiovascular and vascular disorders (VEGF signaling, platelet degranulation, extracellular matrix organization), neurological disorders (axon guidance, cytoskeleton in muscle cells, supramolecular fiber organization), cancer and cell growth dysregulation (insulin-like growth factor, cell migration, VEGF signaling, cell–cell adhesion), protein quality control (vesicle-mediated transport, endocytosis, RHO GTPase, protein folding), and fibrosis (wounding, extracellular matrix, VEGF signaling, cytokine signaling, inflammation).

We then applied our deep hybrid neat plasma spectral library to two of the sample sets of pooled plasma analyzed using the final optimized method: *n* = 16 from the same analysis (from DIA-PASEF window optimization, Figure 2D), and *n* = 54 across three separate plates (from longitudinal reproducibility experiment, Figure 3). We analyzed the data sets in Spectronaut in three different ways: (1) using a classic library-only DIA analysis strategy against the hybrid deep plasma library; (2) using a FASTA-only directDIA search, and; (3) using a mixed-mode search combining a FASTA directDIA search with a deep library extension search. For both sample sets, we found that library-only mode resulted in the fewest identifications at the peptide, precursor, and protein level (Figure 4E,F). For the *n* = 16 data set there were 568 protein groups in total identified with library-only mode compared to 570 with FASTA-only, with 578 compared to 695 for *n* = 54. However, there was a substantially reduced search time required for the classic library-only search, for the *n* = 54 data set just 57 min, compared to almost 16 h for FASTA-only (Figure 4G). In both cases, mixed-mode searching utilizing both the FASTA and hybrid deep plasma library resulted in superior proteome depth (Figure 4E,F). Compared to FASTA-only searching for *n* = 16 there was an 11% increase in precursor coverage corresponding to a 14% increase in both peptide and protein coverage to 652 protein groups. For *n* = 54 there was a 7% increase in precursor and peptide coverage, again translating to a 14% increase in protein coverage to 761 proteins. Compared to the FASTA-only search, mixed-mode searching increased search time slightly, by 82 min for the *n* = 54 experiment (Figure 4G). There was only a small increase in %CV when more proteins were identified in mixed-mode searching was compared to FASTA-only (median %CV 15.8 vs 14.9), and a decrease to 12.8% was observed with library-only searching (Supporting Figure S5).

### Demonstration in Clinical Cohort

Finally, we aimed to demonstrate the end-to-end workflow in a clinical cohort of individual samples. Ninety plasma samples from individuals from the BRICCS coronary artery disease cohort were randomized in case/control order across a 96-well plate, with 6 pooled QC samples placed diagonally across the plate. The samples were prepared using the automated in-solution digestion, peptide cleanup, and automated Evotip loading on the Bravo AssayMAP, and then rerandomized for analysis on the Evosep ONE–timsTOF HT using the final optimized DIA-PASEF method. In this case, FASTA-only searching resulted in the lowest number of hits, with 9004 precursors corresponding to 7536 peptides and 715 proteins (Figure 5A). We expanded our deep library by adding the *n* = 54 replicates of pooled plasma from the longitudinal reproducibility experiment and searched in library-only and mixed mode. Library-only mode had a very low search time of less than 5 h, and identified 11,406 precursors corresponding to 8289 peptides and 845 protein groups. Mixed mode searching again resulted in the highest number of hits, with 11,729 precursors and 8561 peptides resulting in 936 proteins, although with a search time penalty of almost 53 h (Figure 5A). Over 50% of protein groups identified were present using all search strategies, with mixed mode resulting in the greatest addition of unique protein IDs compared to the other strategies (Figure 5B). Thus, the mixed mode search results were used for the subsequent differential expression analysis to confirm the workflow is suitable for detecting biological differences. Proteins identified using stratified single hit protein FDR were responsible for 18.2% of identifications.

**Figure 5:**
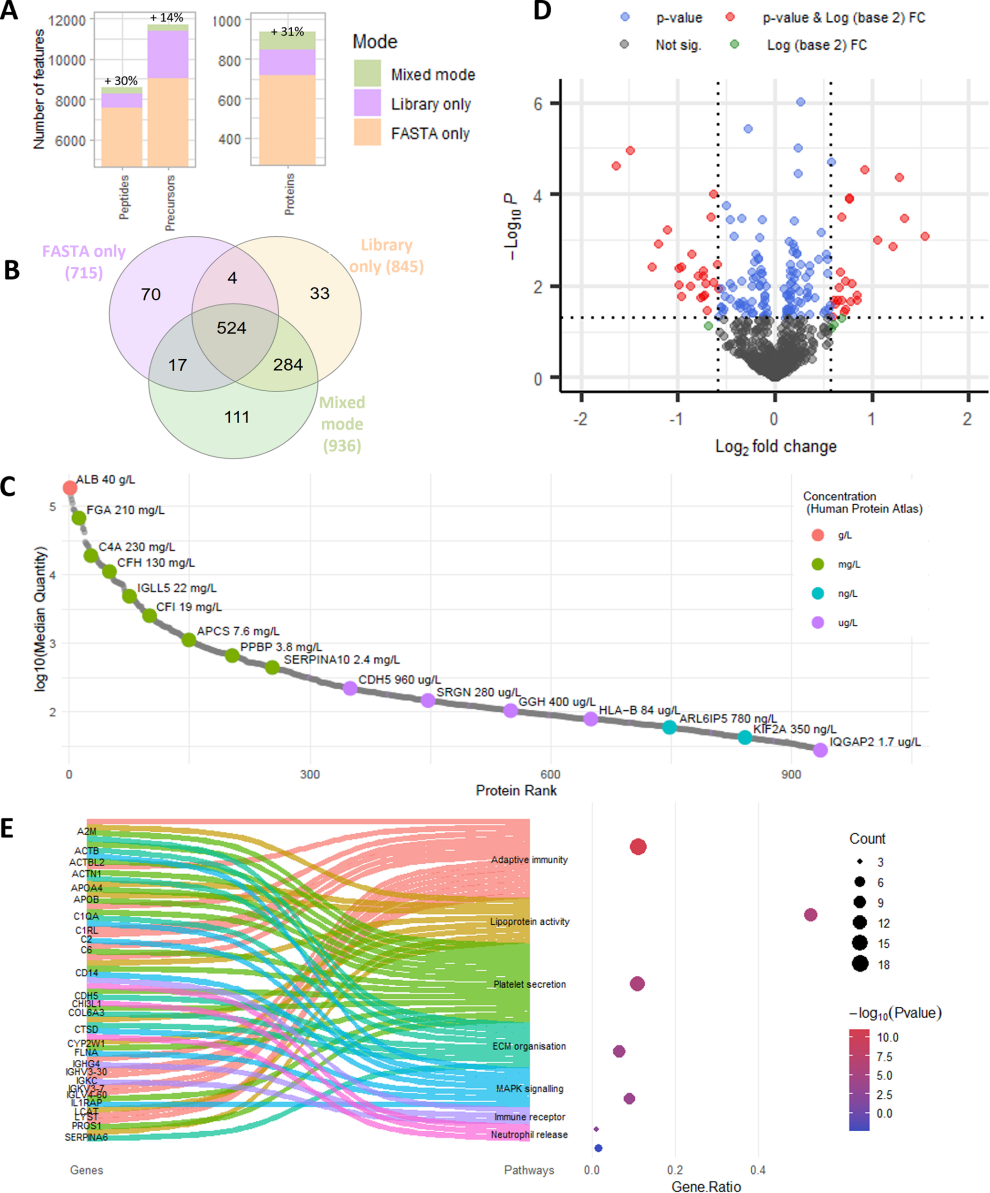
Demonstration of end-to-end workflow in a clinical cohort of 45 individuals with CAD and 45 healthy matched controls (total *n* = 90). (A) The increase in precursor, peptide and protein group IDs over FASTA-only searching when library-only or mixed mode searching is applied to the cohort. (B) Venn diagram showing the overlap in IDs between the different search strategies. (C) The dynamic range of the proteins identified in the CAD cohort, ranked by log_10_(median quantity), with select proteins annotated with their absolute concentrations in blood as recorded on the Human Protein Atlas. (D) Volcano plot showing significant differences between the case group with CAD and healthy controls, with 24 upregulated and 18 downregulated proteins at BH-adj. *p*-value <0.05 and FC > 1.5. (E) Sankey-dot plot showing the relationship between the individual dysregulated genes (left of ribbon) which exhibit the greatest connectivity and enriched biological pathways (right of ribbon). The accompanying dot plot shows the enrichment significance of the pathways.

We assessed the dynamic range of the 936 proteins identified by mixed mode searching (Figure 5C), ranking them by log_10_(median quantity) and comparing this for selected proteins across the range with their absolute blood concentrations recorded in the Human Protein Atlas.^29^ As expected, high-abundance proteins in the g/L and mg/L range and midabundance proteins in the high μg/L range represented most of the proteins identified, However, some lower abundance proteins in the lower μg/L and high ng/L range were also represented. Thus, the proteins identified represented some 8 orders of magnitude in concentration.

We performed differential expression analysis to determine if there were significant differences detected in patients with coronary artery disease compared to the healthy matched controls, and identified with 24 upregulated and 18 downregulated proteins at a BH-adj. *p*-value <0.05 and FC > 1.5 (Figure 5C, Supporting Table S4), the most relevant of which are shown in Figure 5D. The full list of proteins identified with their BH-adj. p-values and log2­(FC) are available in Supporting Information-Protein IDs. We performed pathway enrichment analysis using Metascape^21^ to identify which biological pathways were over-represented by these dysregulated proteins, and found pathways related to adaptive immunity, lipoprotein assembly, platelet and neutrophil degranulation, extracellular matrix (ECM) organization, and mitogen-activated protein kinase (MAPK) signaling to be enriched (Figure 5D). We also combined the significantly upregulated proteins into an equal-weight panel and tested the power of the panel using receiver operating characteristic (ROC) analysis to predict the binary case/control classification, and found that the panel had an area-under-curve (AUC) of 0.979, indicating excellent discriminatory ability for presence of CAD (Supporting Figure S6).

## Discussion

The workflow described herein provides a novel framework for integrating current technologies for maximum benefit in neat plasma proteomics. The fully automated workflow delivers end-to-end sample preparation including in-solution digestion, peptide cleanup, and Evotip loading. Longitudinal reproducibility testing over three independent plates confirmed the robustness of the workflow, with minimal intra- and interplate variation (%CV < 15) and no evidence of positional effects. This stability is critical for clinical applications, where biological variability is high and analytical variability must be minimized.

Through systematic assessment of PASEF windows and library generation, we provide a framework which can be readily applied to other neat plasma studies to accelerate optimization, maximizing proteome coverage while maintaining robustness. We demonstrated that narrowing windows and decreasing ramp/accumulation time improved coverage for neat plasma, while variable window widths (via py_diAID^24^) and diagonal-PASEF increased ppp and reduced %CV, albeit with lower coverage. The final optimized method achieved >700 proteins per sample for pooled neat plasma or >900 proteins for individual donors from a CAD cohort. A broad range of clinically relevant proteins and pathways were captured from neat plasma without depletion, extensive manual fractionation, or large sample volumes, underscoring the clinical feasibility and biomedical value of the workflow. The proteins represented span midto-high abundance ranges, a range in which new panels are most likely to readily translate into robust clinical assays.

In contrast to the OT-2-based protocol previously described for automated Evotip loading,^16^ the Bravo AssayMAP uses high-precision positive pressure for all liquid handling steps. The total time for sample preparation per 96-well plate was equal to 20.6 h assuming an overnight digestion is used, and this can be multiplexed to 4 plates (totalling 384 samples) if the “Multi-Plate” version of the Bravo AssayMAP in-solution digestion protocol is employed. A reduction in digestion time to 4 h, as successfully demonstrated by other authors,^30^ would allow the whole protocol to be performed in 8.6 h. However, a limitation of this study is that we did not assess this. An additional limitation of this workflow is that protein and peptide assays must still be performed off-deck if desired. Automation with the AssayMAP system also enables facile integration of additional processing steps such as phosphopeptide enrichment using TiO_2_ and Fe­(III)-NTA-IMAC,^31^ without needing to change the pipetting head, deck layout, or liquid handling platform. This ease of use is in contrast to the Agilent Bravo autoSP3 protocol which, although powerful, would require switching pipetting heads to load Evotips, thus interrupting the workflow.^32^

We also extended the platform with a novel application of TIMS gas-phase fractionation (GPF), an approach that has been applied successfully to HeLa digests and depleted plasma but, to our knowledge, never to undepleted plasma.^8,9^ A deep hybrid library was built from fully automated high-pH RP fractionation and multiple previously published DIA- and DDA-PASEF GPF schemes, which contained 1788 protein groups.^7,8,22^ Notably, high-pH RP fractionation, often a labor-intensive and error-prone offline process, was fully automated on the same platform used for digestion and Evotip loading, ensuring consistent handling and eliminating manual steps that could introduce variability. We demonstrated that integrating the deep hybrid library into data analysis via mixed-mode searching (FASTA + library) boosted protein group coverage by up to 31% compared with FASTA-only searches, without adversely affecting reproducibility. A limitation of this workflow is that mixed-mode searching resulted in a modest increase in search time compared to FASTA-only. High-performance computing (HPC) infrastructure will likely play an increasingly important role as proteomics studies grow larger in sample size, enabled by automated sample preparation and high-throughput instruments. Indeed, van Zalm et al. previously reported a HPC parallelization strategy with FragPipe which reduced the total runtime by ∼90% in the analysis of 3348 plasma samples.^33^

It is worth noting that there were unique proteins identified by each of the search strategies. In a library-only search, the strength and depth of the library can influence search outcomes, whereas FASTA-only searches may achieve greater coverage in protein groups not represented by the library. In a mixed-mode search strategy, changes in score competition and FDR modeling can suppress IDs close to the FDR-threshold which appeared in the other searches; these may represent borderline but genuine IDs, or false positives. This highlights the importance of careful evaluation of the appropriate search strategy on a case-by-case basis, and that further validation of discovery hits should be performed using, for example, a targeted proteomics assay. Further validation should also be performed where single-hit proteins are used for identification. Spectronaut by default employs a stratified single-hit protein FDR, meaning that single-peptide proteins are evaluated separately during global protein-level FDR estimation. This imposes more stringent FDR control on single-hit proteins, but careful interpretation is still needed.

Finally, we demonstrated the end-to-end workflow in a clinical cohort of CAD patients and healthy matched controls. Using mixed-mode searching, 936 proteins were identified, 42 of which were dysregulated in CAD. Enriched pathways in the BRICCS CAD cohort were identified which are reflective of an atherosclerotic state, including adaptive immunity,^34^ dysregulated lipoprotein assembly,^35^ platelet and neutrophil degranulation,^36^ ECM organization,^37^ and MAPK signaling.^38^ This underscores the biological relevance of the findings, and demonstrates the utility of the workflow for biomarker discovery. Assessment of the dynamic range of the proteins identified showed that, as expected, most detected proteins were high- or midabundance species present in the g/L to μg/L range. Nevertheless, a subset of lower-abundance proteins in high ng/L range were also identified, demonstrating that the workflow can capture some lower abundance proteins. However, a limitation of this approach which focuses on midto-high abundance proteins is that many molecules of value to novel biomarker and mechanistic studies are not captured in the analysis, including cytokines and signaling molecules such as interleukins, tumor necrosis factors, and chemokines. It is possible that incorporating targeted proteomic assays (multiple reaction monitoring or parallel reaction monitoring) coupled with nanoLC separation could enable detection of these ultralow-abundance proteins in neat plasma.

Despite these limitations, the proteome depth achieved compares favorably to other established workflows for neat plasma, achieving better proteome depth for a comparable throughput of 100 SPD, or similar depth at higher throughput.^13,16,22^ Distler et al. performed a thorough multicenter evaluation of a high dynamic range human plasma benchmark set including 21 different instrument configurations spanning nine different mass spectrometers.^39^ For *n* = 6, six configurations exceeded the 359 protein groups identified with our final optimized method. However, these methods utilized lower throughput (ranging from 29–102 min, the equivalent of 48–14 SPD). One analysis was performed using 100 SPD throughput with the nanoAcquity LC coupled to the timsTOF Pro, which identified 229 proteins, comparatively lower to our optimized method on the Evosep ONE–timsTOF HT.

Our workflow makes use of an Agilent Bravo, an Evosep ONE, and a timsTOF HT, but the general principles are transferable to other robotic platforms. The steps required for reduction, alkylation, and digestion can be readily applied to other pipetting robots such as the Andrew + or Opentrons OT-2, which also have their own compatible C18 SPE cartridges or plates available for cleanup. In the case of the Opentrons OT-2, automated Evotip loading can also be performed on-deck.^16^ The DIA-PASEF optimizations described for the timsTOF HT are inherently transferable to other timsTOF platforms such as the Ultra 2, Pro 2, and SCP. For other platforms, the broader considerations are applicable such as library generation, as well as balancing cycle time, LC peak width, data points per peak, and proteome coverage, including other LCs and Orbitrap-based systems. With the advent of the Evosep ENO, which boasts better peak capacity, narrower XIC widths, and significantly better performance at gradients up to 500 SPD, the trade-offs discussed in this work of cycle time, data points per peak, and proteome coverage will become even more relevant.

The workflow demonstrated offers a reproducible, sustainable, and scalable platform for neat plasma proteomics, combining automation of all key preparation steps with acquisition and data processing strategies that maximize coverage while preserving quantitative robustness. The ability to incorporate both online and fully automated offline fractionation, generate deep disease-relevant libraries, and maintain longitudinal reproducibility positions this workflow as a powerful tool for large-scale biomarker discovery and clinical proteomics.

## Supplementary Material







## Data Availability

The raw mass spectrometry data and libraries generated in this study have been deposited in the MassIVE repository under accession number MSV000099480.

## Notes

Supporting Information includes supporting Figures and Tables (Supporting Information.docx) (PDF)
PASEF methods (Supporting Information-timsTOF HT methods.xlsx) (XLSX)
protein IDs for each experiment (Supporting Information-Protein IDs.xlsx) (XLSX)
