# Supplementary material for: Harnessing New Tools for Old Challenges: Optimising Neat Plasma Proteomics with Automation and Gas-Phase Fractionation
Source: ACS Meas Sci Au. 2026 Jan 5;6(1):224–37. doi: 10.1021/acsmeasuresciau.5c00166 (PMC12921592; doi:10.1021/acsmeasuresciau.5c00166)
Supplement: Supplementary file 1 [file tg5c00166_si_001.pdf]

Harnessing New Tools for Old Challenges: Optimising Neat Plasma Proteomics with Automation and Gas-Phase Fractionation

Supporting Information

Colleen B. Maxwell[1,2*], Dan Lane[1,2], Nikita Bhakta[1,2], Emer M. Brady[1], Richard D. Haigh[3], Rajinder Singh[1,2], Gaurav S. Gulsin[1], Gerry P. McCann[1], Leong L. Ng[1,2], Donald J. L. Jones[2,4]

1. Division of Cardiovascular Sciences and NIHR Leicester Cardiovascular BRC, Glenfield Hospital LE3 9QP, University of Leicester, Leicester, UK
2. Leicester van Geest MultiOMICS Facility, Hodgkin Building LE1 9HN, University of Leicester, Leicester, UK
3. Leicester Drug Discovery and Diagnostics (LD3), Maurice Shock Medical Sciences (MSB) LE1 7RH, University of Leicester, UK
4. Division of Cancer Sciences, Robert Kilpatrick Clinical Sciences Building, Leicester Royal Infirmary LE1 5WW, Leicester, UK

*Corresponding author cbm11@le.ac.uk

*Corresponding author cbm11@le.ac.uk

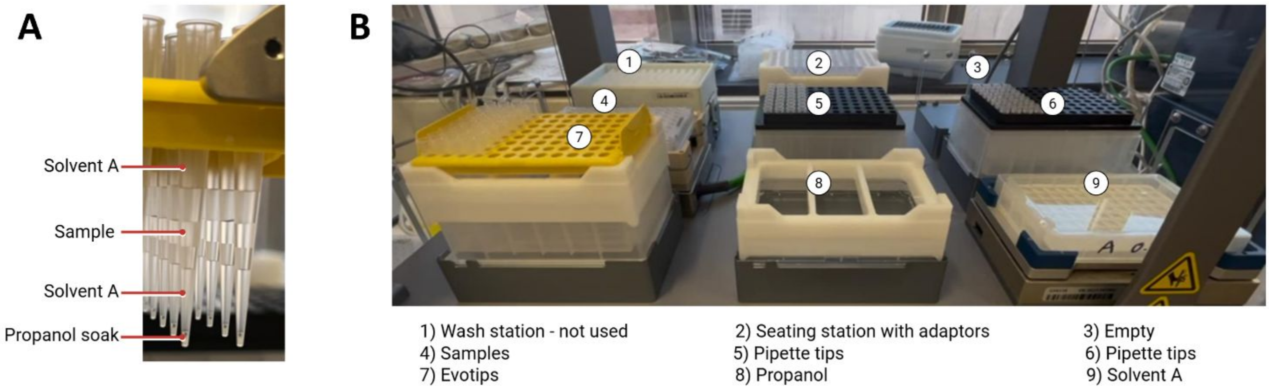

Supporting Figure 1. Fully Automated Evotip Loading. **(A)** Liquid levels formed automatically during the protocol preceding positive air pressure dispensing the liquid through the sorbent bed**. (B)** Deck layout of the fully automated Evotip loading protocol.

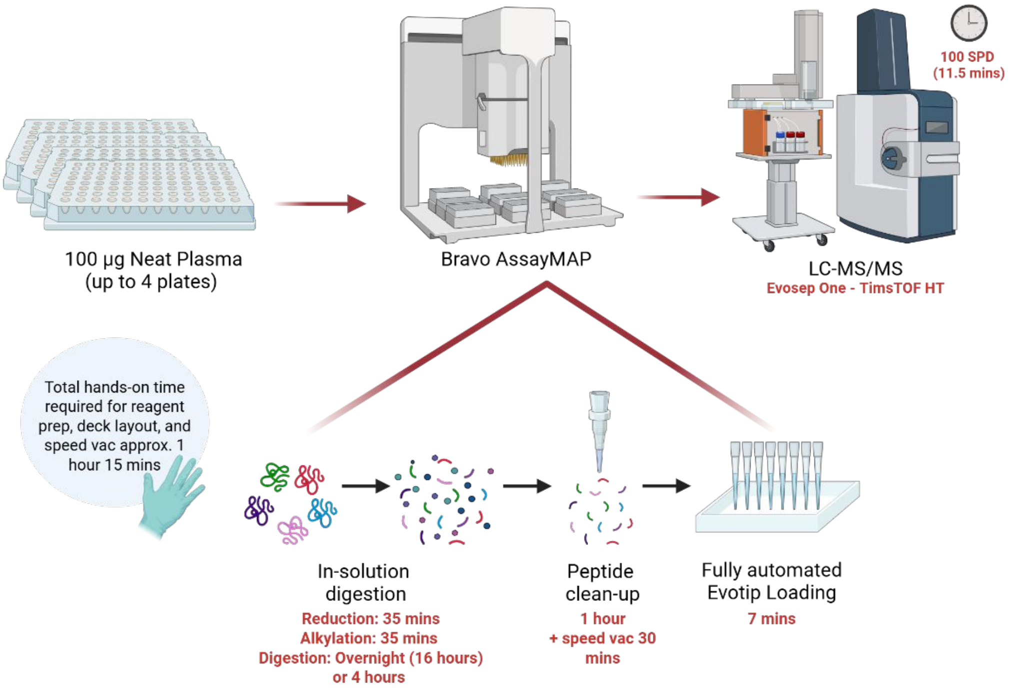

Supporting Figure 2. Workflow diagram showing the time taken to complete the end-to-end workflow comprising in-solution digestion, peptide clean-up, fully automated Evotip loading, and LC-MS/MS analysis. Sample preparation would require approx. 20 hours for overnight digestion, or 8.6 hours for a shorter digestion time. Assuming the plasma plates have already been prepared, some hands-on time is also required for each step, including reagent preparation and aliquoting reagents into plates (approx. 1 hour), setting up the deck for digestion (3 mins), setting up the deck again for peptide clean-up (5 mins), transfer of the plate to the speed vac and reconstituting for Evotip loading (5 mins), deck set-up for Evotip loading (2 mins). Created in BioRender *https://BioRender.com/q7hzgjb*.

Supporting table 1. Baseline characteristics of the two matched groups from the BRICCS cohort (control CACS <10 and case CACS ≥ 200).

Characteristic		Control n=45	Case n=45
**Age (at CT scan) (mean (SD))**		61.46 (6.34)	61.56 (6.42)
**BMI (mean (SD))**		29.22 (5.13)	29.61 (5.89)
**Average Systolic Blood Pressure (SBP) (mean (SD))**		134.36 (15.44)	138.58 (20.58)
**Average Diastolic Blood Pressure (DBP) (mean (SD))**		83.26 (10.59)	84.78 (11.69)
**Sex = Male (%)**		38 (76.0)	38 (76.0)
**Ethnicity = non-Caucasian (%)**		9 (18.0)	12 (24.0)
**Smoking = Ever (%)**		25 (50.0)	22 (44.0)
**Smoking Status (%)**	**Non**	22 (44.0)	28 (56.0)
	**Ex**	17 (34.0)	18 (36.0)
	**Current**	8 (16.0)	4 (8.0)
**History of High Blood Pressure = Yes (%)**		24 (48.0)	24 (48.0)
**Diabetes = Yes (%)**		9 (18.0)	9 (18.0)

Supporting table 2. Summary of the parameters and results of the timsTOF HT PASEF methods trialled with the Evosep Endurance column.

Method	Default	Default Short	Optimised 1	Optimised 2	Py_diAID	Diagonal
**Mass range (*m/z*)**	400 - 1000	475 - 1000	392 - 1027	392 - 1018	300 - 1199	400 - 980
**Mobility range (1/K_0)**	0.64 - 1.37	0.85 - 1.27	0.67 - 1.3	0.67 - 1.3	0.65 - 1.35	0.82 - 1.25
**Mass width (Da)**	25	25	32	25	Variable	30
**Number of MS/MS ramps**	8	8	10	15	8	3
**Number of MS/MS windows**	24	21	20	25	16	NA
**Ramp/accum time (ms)**	100	100	100	100	80	80
**Cycle time (s)**	0.95	0.95	1.17	1.7	0.77	0.42
**Precursors**	3597	2874	3797	4002	3782	3276
**Peptides**	2891	2462	3072	3274	2876	2572
**Proteins**	288	290	302	326	272	261
**Median datapoints per peak**	7	7	6	6	8	11
**Mean datapoints per peak**	6.92	6.69	6.24	5.78	8.01	11.3
**Median %CV**	7.4	9.6	8	8.5	8.4	6.7
**Data completeness**	97	96.6	97.3	96	95.7	97.2
**Median XIC width (min)**	0.9	0.8	1	1	0.9	1.1

Supporting table 3. Summary of the parameters and results of the timsTOF HT PASEF methods trialled with the Evosep Performance column.

Method	Default	Optimised (100 ms)	Optimised (80 ms)	Optimised (60 ms)	Final Optimised (50 ms)	Diagonal
**Mass range (*m/z*)**	400 - 1000	392 - 1018	392 - 1018	392 - 1018	392 - 1018	400 - 980
**Mobility range (1/K_0)**	0.64 - 1.37	0.67 - 1.3	0.67 - 1.3	0.67 - 1.3	0.67 - 1.3	0.82 - 1.25
**Mass width (Da)**	25	25	25	25	25	30
**Number of MS/MS ramps**	8	15	15	15	15	3
**Number of MS/MS windows**	24	26	26	26	26	NA
**Ramp/accum time (ms)**	100	100	80	60	50	80
**Cycle time (s)**	0.95	1.7	1.38	1.06	0.9	0.42
**Precursors**	4150	4239	4106	4189	4618	4377
**Peptides**	3298	3393	3258	3277	3562	3229
**Proteins**	320	329	321	335	359	316
**Median datapoints per peak**	4	3	3	4	5	8
**Mean datapoints per peak**	4.71	3.1	3.68	4.37	5.15	9.1
**Median %CV**	8.5	8.7	7.8	8.4	7.8	6.6
**Data completeness**	96.3	94.8	96.7	98.9	97.4	94.2
**Median XIC width (min)**	0.6	0.6	0.5	0.52	0.6	0.6

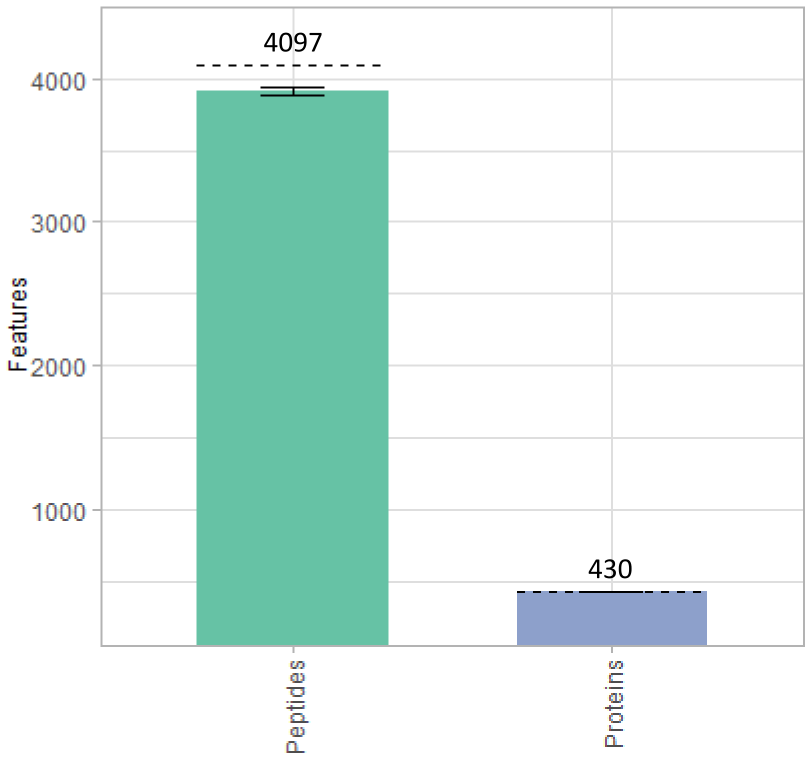

Supporting Figure 3. Mean number of peptides and proteins groups identified in each sample for 60 SPD ± σ for n=6 pooled plasma samples. Total proteins indicated by dashed line.

Supporting table 4. Summary of the parameters and results of the timsTOF HT PASEF methods trialled with the Evosep Performance column.

Method	100 SPD	60 SPD	% Increase in 60 SPD
**Gradient length (mins)**	11.5	21	82.6
**Total injection cycle time (mins)**	14	24	71.4
**Precursors**	4618	5307	14.9
**Peptides**	3562	4097	15.0
**Proteins**	359	430	19.8
**Median datapoints per peak**	5	6	20
**Mean datapoints per peak**	5.15	6.7	30.1
**Median %CV**	7.8	9.7	24.4
**Data completeness**	97.4	96.2	-1.23
**Median XIC width (min)**	0.6	0.8	33.3

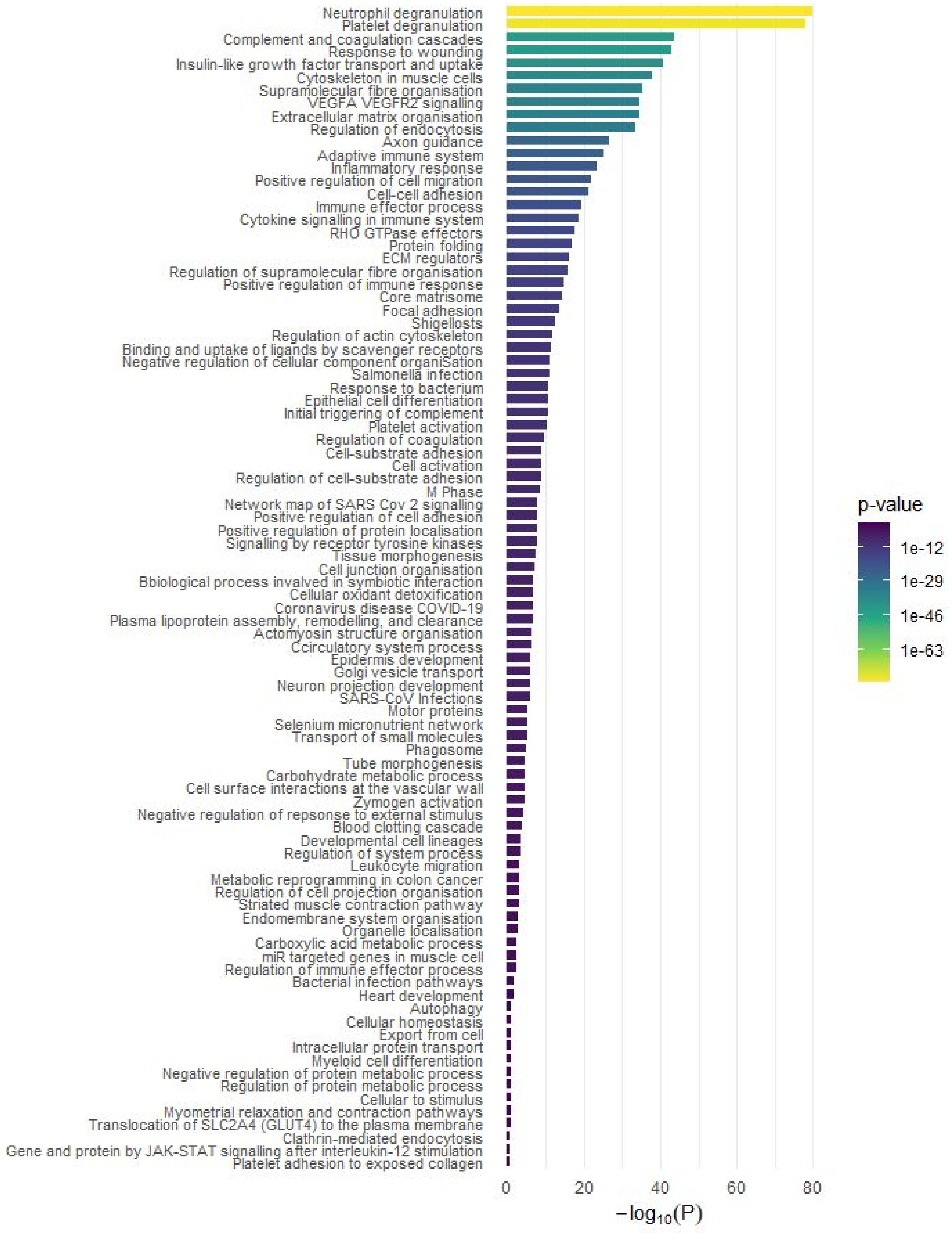

Supporting Figure 4. Bar chart showing top 90 biological pathways which are over-represented in the deep plasma library alongside their -$\log_{10}$(P-values).

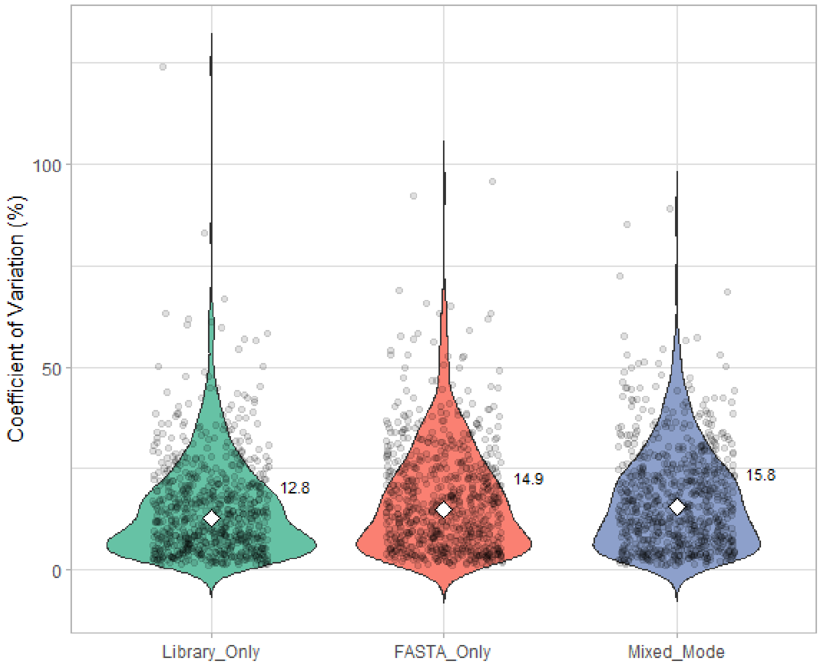

Supporting Figure 5. Violin plot of the % CV distribution across n=54 samples analysed using library-only mode (green), FASTA-only mode (pink), and mixed-mode searching (blue) employing the deep hybrid plasma library. The median CV is indicated with the white marker and is annotated to the right of each violin.

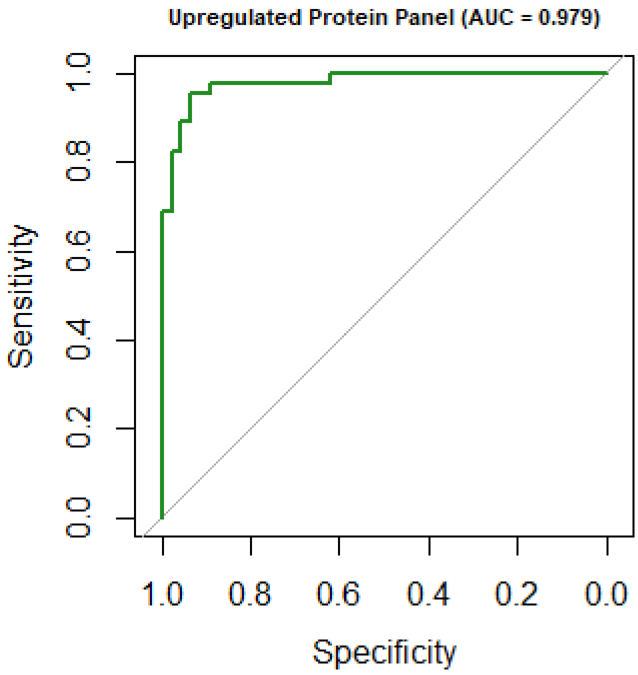

Supporting Figure 6. Receiver Operating Characteristic (ROC) curve of the significantly upregulated proteins combined into an equal-weighted panel in the BRICCS sub-cohort showing sensitivity (%) vs. specificity (%) with an AUC of 0.979.

Supporting table 5. Differentially expressed proteins, defined by <0.05 adj.p and >1.5 absolute fold-change, between coronary artery disease (CAD) and control in the BRICCS sub-cohort sorted by p-value (lowest – highest) with Benjamini-Hochberg adjusted p-values and fold change (FC).

Protein	BH adj. p-value	FC
**CD14**	0.0006	1.50
**IGKV3-7**	0.0012	-1.51
**A2M**	0.0019	-2.82
**MTMR14**	0.0019	1.68
**IWS1**	0.0025	1.89
**LYST**	0.0025	-3.12
**IGHV3-23**	0.0028	1.89
**SERPINA6**	0.003	1.88
**EMC1**	0.0032	2.44
**IGHG4**	0.0066	-1.55
**IGLV4-60**	0.0069	1.70
**CHI3L1**	0.0071	1.70
**FLNA**	0.0088	-1.52
**SMIM18**	0.0121	-1.7
**CTSD**	0.0121	-1.58
**IGHV3-30**	0.0121	-1.98
**IGKC**	0.0121	-1.76
**IL1RAP**	0.0121	1.62
**SAR1A**	0.0121	1.95
**IGLV2-18**	0.0121	2.53
**ANKDD1A**	0.0188	-2.15
**APOA4**	0.0205	1.59
**PTAR1**	0.0225	-1.74
**ACTN1**	0.0225	2.92
**PROS1**	0.0227	-1.52
**COL6A3**	0.0246	2.08
**LCAT**	0.0259	1.51
**CDH5**	0.0281	-2.30
**CYP2W1**	0.0281	1.54
**CRISP3**	0.0296	2.33
**C2**	0.0304	1.54
**C1RL**	0.0355	1.54
**IGLV2-11**	0.0368	-1.81
**IGHV3-15**	0.0368	-1.64
**APOB**	0.0368	1.66
**ACTBL2**	0.0368	1.67
**IPMK**	0.0368	2.04
**C6**	0.0418	-1.63
**SFTPB**	0.0419	-1.53
**ARF3**	0.0419	1.97
**ACTB**	0.0419	1.86
**C1QA**	0.0477	-1.66
